# Extracorporeal shockwave treatment in knee osteoarthritis: therapeutic effects and possible mechanism

**DOI:** 10.1042/BSR20200926

**Published:** 2020-11-13

**Authors:** Senbo An, Jingyi Li, Wenqing Xie, Ni Yin, Yusheng Li, Yihe Hu

**Affiliations:** 1 Department of Orthopaedics, Shandong Provincial Hospital Affiliated to Shandong First Medical University, Jinan, Shandong, P.R. China 250021; 2 Department of Orthopaedics, Xiangya Hospital of Central South University, Xiangya Road 87, Changsha, Hunan, P.R. China 410008; 3 Department of Clinical Medicine (8-Year Program), Xiangya Medicine School, Central South University, Changsha, Hunan, P.R. China 410013; 4 National Clinical Research Center for Geriatric Disorders, Xiangya Hospital, Central South University, Changsha, Hunan, P.R. China 410008

**Keywords:** extracorporeal shockwave, osteoarthritis, pain, subchondral bone

## Abstract

Osteoarthritis (OA), the most common degenerative joint disease, is characterized by the cardinal symptoms of chronic pain and restricted joint activity. The complicated pathological changes associated with OA and unclear mechanistic etiology have rendered existing non-surgical OA management options unsatisfactory. Increasing clinical and experimental evidence suggests that extracorporeal shockwave therapy (ESWT) is beneficial in OA treatment. ESWT is found to have modifying effects on cartilage and subchondral bone alterations in OA progression, as well as the clinical complaints of patients, including chronic pain and limited joint activities. However, the specific treatment strategy regarding the dosage and frequency of ESWT is still underdetermined. This review discusses the existing evidence regarding the therapeutic indications and possible mechanism of ESWT for OA treatment.

## Introduction

Osteoarthritis (OA), which is often described as a degenerative joint disease, is the most common form of arthritis, followed by rheumatoid arthritis (RA), gout and lupus [1]. The cardinal symptoms of OA include pain, transient morning stiffness, a grating sensation in the joint and a loss of function that may ultimately lead to instability and physical disability and an impaired quality of life, which places heavy burdens on the affected individuals and their communities [2,3].

The treatment of OA remains challenging, and the exact definition, risk factors and pathophysiology of the disease remain incompletely elucidated [4,5]. OA pain is the primary complaint expressed by patients who seek clinical intervention. Multiple therapies, including pharmacological and non-pharmacological interventions, have been applied to patients with OA [6]. However, none of these treatments can completely eliminate OA pain.

In the past 15 years, extracorporeal shockwave therapy (ESWT) has emerged as a leading option for the efficient treatment of musculoskeletal disorders such as tendinopathy, lateral epicondylitis, calcific tendinitis and non-union of long bone fracture, as well as avascular necrosis of the femoral head [7–10]. In the United States, the Food and Drug Administration (FDA) has approved several specific shockwave devices for the treatment of proximal plantar fasciitis and lateral epicondylitis of the elbow [11,12]. In recent years, ESWT has been considered and introduced in the treatment of OA and investigations have demonstrated that ESWT can ameliorate the pathologic changes of OA, including cartilage and subchondral bone changes [13].

Recent studies have shown that ESWT can accelerate the healing of meniscal degeneration and plays a chondroprotective role in OA [14–16]. ESWT treatment can increase the activity of chondrocytes and decrease cartilage fissuring, as well as chondrocyte apoptosis [17], and it also has been proved that the chondroprotective effect is consistent and beneficial both in early or later stage of OA [18,19]. Furthermore, ESWT treatment can alleviate OA pain and improve motor function both in animal models and clinical trials [7,20,21]. These reports indicate the potential clinical application of ESWT as a novel treatment for OA.

At present, evidence show that definite effectiveness of ESWT in OA remains insufficient. Encouraged by the benefit of ESWT treating other musculoskeletal diseases, the purpose of the present study is to provide a positive overview in ESWT treating OA. We collected evidence from clinical trials and animal model studies to explore the function and possible mechanism of ESWT as a potential treatment for OA.

## Extracorporeal shockwave

Extracorporeal shockwave can be generated using an electrohydraulic, electromagnetic or piezoelectric source, and each source possesses features that can address specific diseases [22]. The first-generation shockwave machine used for musculoskeletal disease therapy was based on an electrohydraulic source that generated high-energy acoustic waves through an underwater explosion with a high-voltage electrode spark discharge. The shockwave energy output was transmitted through an elliptical reflector with a large-axial-diameter focal volume, and targeted at the diseased area of the body [23].

An electromagnetic source can generate a shockwave by passing an electric current through a coil to produce a magnetic field, which results in a sudden deflection of the membrane and generation of pressure waves in a fluid. Subsequently, a lens is applied to focus these waves onto the diseased area of the body, and the length of the lens can be used to determine the therapeutic point [24]. Piezoelectric sources can produce shockwaves via a high-voltage discharge across piezoelectric elements (i.e., a large number (>1000) of piezo crystals) mounted in a sphere, which induces a pressure pulse in the surrounding water that increases to a shockwave. The expansion of each element generates a pressure pulse that can enable the self-focusing of waves toward the target, leading to an extremely precise focus and high level of energy within a focal volume. The different features of these shockwave devices, including pressure distribution, energy density and total energy at the second focal point, can be used to treat varied diseases such as urolithiasis and musculoskeletal disorders.

In contrast with an ultrasound wave, a shockwave is uniphasic, with a peak pressure as high as 500 bars (∼1000 times the pressure of an ultrasound wave) [23]. Despite its successful clinical application, the mechanism of ESWT remains unclear. Possible physical, physicochemical, chemical and biological mechanisms underlying the effects of ESWT on tissues have been identified [25]. In the physical phase, a shockwave induces a positive pressure and thus the absorption, reflection, refraction and transmission of energy to tissues [23]. In the physicochemical phase, ESWT stimulates cells to release biomolecules such as adenosine triphosphate (ATP) and thus activate signaling pathways such as the extracellular signal-regulated kinase (ERK), focal adhesion kinase (FAK) and Toll-like receptor 3 (TLR3) pathways [25–27]. In the chemical phase, shockwaves can mediate transmembrane cellular ion channels and intracellular calcium flux [28]. Finally, previous studies have demonstrated several biological effects of ESWT, including improved angiogenesis, wound healing and bone non-union healing; modulation of tissue and nerve regeneration and inhibition of inflammatory activities [29–32].

**Figure 1:**
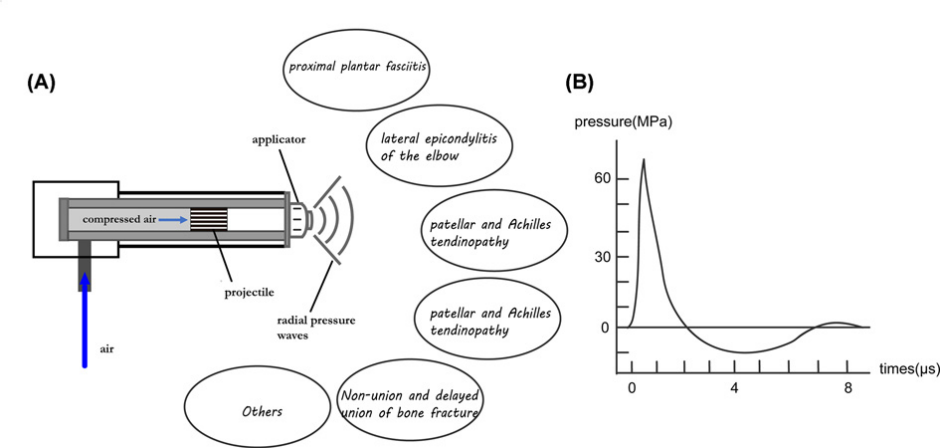
Illustrations of ESWT generated by radial pressure wave source (**A**) and its characteristics (**B**)

Studies have proved ESWT can treat bone- and muscle-related diseases. Most clinical studies that have investigated the efficacy of ESWT for the treatment of proximal plantar fasciitis [33–36] and lateral epicondylitis of the elbow [37–39] have reported promising results and negligible complications. Many studies of ESWT for the treatment of patellar tendinopathy and Achilles tendinopathy have also yielded favorable results [40,41]. Reports of ESWT for the non-union and delayed union of long bone fracture have demonstrated the successful achievement of bone unions [42,43]. ESWT was also recently applied to the treatment of avascular necrosis of the femoral head [44]. Other reports described the positive effects of ESWT on knee OA [18], spinal fusion [45] and chronic diabetic foot ulcers [46]. However, the exact mechanism by which shockwave therapy mediates these effects remains unclear. We here showed a diagram of ESWT generated by radial pressure wave source and the characteristic of the wave source (Figure 1) referring to a study by Moya et al [47].

Air is draw in and compressed in the tube, which could cause a sudden rise of positive pressure towards the projectile, forming a radial wave toward the target through the applicator. ESWT can be applied to treat several musculoskeletal disorders. After the peak positive pressure, a negative pressure is formed and forced the projectile back, being ready for the next wave cycle.

## Extracorporeal shockwave treatment for osteoarthritis

Very recently, animal model studies have increasingly demonstrated the efficacy of ESWT for OA [16,17,19]. However, few studies have explored the clinical application of ESWT, probably due to a lack of mechanistic clarity and scientific evidence [48]. OA is characterized by the main features of articular cartilage degeneration and subchondral bone remodeling and cardinal symptoms of pain and restricted motion in the affected joints. Several studies [15,16,49] have proven that ESWT may have anti-inflammatory, angiogenic, anti-edema and trophic effects in the modification of cartilage and subchondral bone, the most featured pathological changing sites in OA [50].

Liu et al. [51] investigated the efficacy of ESWT combined with hyaluronic acid (HA) in patients with knee OA and used scoring scales, including a visual analog scale (VAS), Western Ontario and McMaster Universities Osteoarthritis Index (WOMAC) and knee injury and osteoarthritis outcome score (KOOS), to demonstrate the superiority of the combined approach relative to HA alone, since single HA treatment still has limited efficacy and accompanies a couple of adverse events [52]. An *in vitro* study [53] explained that ESWT could increase the activity of chondrocytes through inducing the surface expression of major hyaluronan cell receptor CD44, which could allow the increase of Collagen type 2A/ Collagen type 1A (COL2A/COL1A) ratio when using HA, and this could enhance the cell susceptibility of chondrocytes to HA, favoring the repair of degenerated cartilage. Another randomized controlled trial showed that ESWT can improve WOMAC and range of motion (ROM) better than kinesiotherapy (KIN) on knee OA [54]. Further, a retrospective study [49] showed that compared with laser therapy, patients underwent ESWT showed greater effect in symptoms relief with regard to WOMAC and Numeric Rating Scale (NRS) evaluation, while no adverse events occurred in both groups. These findings indicated that ESWT could reverse OA progression and ameliorate the associated pain.

### Effect of ESWT on cartilage

Cartilage destruction is one of the most obvious changes associated with OA. Wang et al. [18] demonstrated a significant decrease in articular cartilage degradation via safranin-O staining and Mankin scores after the application of ESWT with 800 impulses at 14 kV for the treatment of OA in a rat model of anterior cruciate ligament transection (ACLT). Interestingly, they assessed pathological changes of cartilage on different locations of the knee after ESWT in early stage OA in Rats. They found ESWT treated medial femur and tibia condyles had been proved better than medial tibia condyle, medial femur condyle as well as medial and lateral tibia condyles in gross osteoarthritis areas, osteophyte formation, thickness of calcified and un-calcified cartilage analysis, cartilage damage and proteoglycan loss.

In rats with knee OA, ESWT was proved to reduce the extent of common disease manifestations, such as cartilage and matrix degradation, as well as the levels of C-telopeptide of type II collagen (CTX-II) and matrix metalloproteinases (MMPs) [17,30]. ESWT may also exert chondroprotective effects in OA models by restoring the production of IL-10 and TNF-α in chondrocytes to normal levels [55]. Kim et al. [48] investigated effects of ESWT on temporomandibular joint osteoarthritis (TMJOA) in Rat OA models, and found ESWT treatment could increase cell viability significantly and decrease expression of cartilage degradation markers including MMP3 and MMP13. Changes in cytochrome *c* and cleaved caspase-3 levels relative to procaspase-3 were also decreased by ESWT, which showed a significant decrease in pro-inflammatory and cartilage degradation markers.

Additionally, Hashimoto et al. [56] suggested that ESWT could accelerate the healing of meniscal tears in avascular regions that might contribute to OA progression in a rat model. Specifically, ESWT promoted the healing of avascular tears by stimulating the proliferation of meniscal cells and the up-regulation of cartilage-repairing factors, including CCN2, SOX9, aggrecan and Col2a1, leading to the increased production of cartilage-specific extracellular matrix.

### Effect of ESWT on subchondral bone

Studies have shown that uncoupled remodeling in the subchondral bone is a critical contributor to the imbalance of mechanical forces and pressure distributions within the knees, which then damage cartilage over the surfaces of subchondral bones [50,57]. Several reports have described the importance of subchondral bone during early-stage OA and suggested that subchondral bone modification may represent a therapeutic target in OA treatment [58,59].

Other studies observed a decrease in the subchondral trabecular bone mass in a rat model of ACLT without ESWT, whereas ESWT targeted at points below the medial tibia led to a considerable increase in osteocytes [19]. These results suggest that ESWT may promote the treatment of OA by enhancing anabolism and improving the subchondral bone microarchitecture. However, patients with late-stage OA experience subchondral bone sclerosis and osteophyte formation subsequent to early bone losses, and thus ESWT may induce osteophytosis, a detrimental condition, at later stages [60].

Chou et al. [16] compared the effects of ESWT on the subchondral bone and articular cartilage in a rat model of early-stage knee OA. Notably, rats subjected to medial menisectomy and ACLT [T(M) group] treated by ESWT showed significantly promoted in pathological examination, micro-CT analysis and cartilage grading score compared with groups underwent ACLT and menisectomy without ESWT. An immunohistochemical analysis revealed significantly up-regulated TGF-β1 expression and down-regulated DMP-1, MMP-13 and ADAMTS-5 in cartilage specimens from the T(M) group, suggesting that subchondral bone was preferable to articular cartilage as a target for ESWT in this model. Chen et al. [61] demonstrated that ESWT could promote the expression of TGF-β1 and VEGF and thus enhance bone regeneration by recruiting mesenchymal stem cells in the bone marrow.

### Amelioration of knee OA pain by ESWT

As noted previously, OA pain is the primary reason patients seek clinical treatment. Studies have indicated that continuous nociceptive input from the osteoarthritic joint drives sensitization of both the central and peripheral nervous systems. Normally, nerve nociceptors are localized within specific tissues and can only detect sensations in the designated area. During OA progression, however, the increased levels of cytokines, chemokines, inflammatory factors, mechanical stimuli and innervation act to expand the nociceptive input [62]. This expansion can lead to hypersensitivity with exaggerated pain (hyperalgesia) in response to noxious stimuli or even innocuous stimuli that are perceived as painful (allodynia) [63]. Chronic pain that cannot be controlled completely by pharmacologic or non-pharmacologic treatment may lead patients to avoid activities and seek protective immobilization. Therefore, pain management is the main therapeutic goal during the treatment of knee OA.

Chronic OA pain appears to be related closely to an increase in the growth of nonvascular, substance P-positive sensory nerve fibers [64]. Many studies have demonstrated the ability of ESWT to alleviate OA pain in animal models. This beneficial effect has been attributed to the promotion of neovascularization, which could increase the blood supply and repair inflamed tissues via tissue regeneration. In a previous study, Ochiai et al. [65] applied treatment to the medial sides of knees in a rat model of OA. Subsequently, a histomorphologic analysis of tissues from ESWT-treated rats revealed a significant decrease in the levels of calcitonin gene-related peptide (CGRP) in the dorsal root ganglia (DRG) neurons, which are closely involved in the sensation of joint pain. ESWT is thought to inhibit the upward transmission of pain via sensory nerve fibers by degenerating the nerve endings [66]. This degeneration would help to reduce the number of CGRP-positive DRG neurons and thus relieve OA pain. Additionally, Revenaugh et al. [67] used ESWT to treat horses with carpal, tarsal or metatarsal OA and applied flexion tests to evaluate pain levels in the OA joint. Notably, the animals treated with ESWT exhibited mild to moderate responses to flexion tests, whereas those in the control group exhibited moderate to severe responses. Chen et al. [20] conducted a randomized, controlled study to compare the effects of ESWT and ultrasound therapy on knee OA with popliteal cyamella. Patients were randomly assigned to four groups. Groups 1–3 received isokinetic muscular strengthening exercises for 8 weeks. Group 2 received tr pulse ultrasound therapy, while Group 3 received weekly ESWT for 8 weeks. Group 4 acted as a control. The effects of the indicated therapies were evaluated after treatment and at a 6-month follow-up using the arthritic knee range of motion (ROM), VAS, Lequesne’s index and peak muscle torque. Only Groups 2 and 3 exhibited significant improvements in ROM after treatment, whereas only Group 3 exhibited an immediate improvement in ROM, as well as the greatest increase in muscle strength after treatment and follow-up. These results suggest that ESWT is superior to pulse ultrasound therapy in terms of pain relief in patients with knee OA and popliteal cyamella.

## Conclusions and perspective

**Figure 2:**
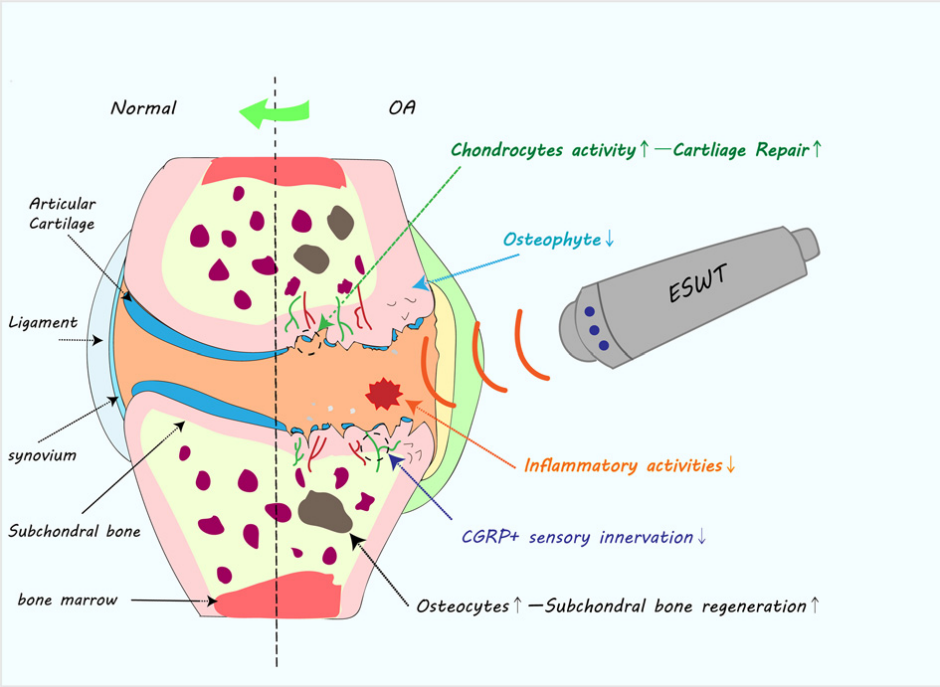
Diagram of positive effect of ESWT on treating OA ESWT could possibly promote the cartilage regeneration by activating chondrocytes, and decrease the number of osteophytes; it can also increase osteocytes activity and decrease the CGRP^+^ sensory nerve fibers in the subchondral bone; besides, ESWT could alleviate chronic inflammatory activities in the whole joint through down-regulating inflammatory cytokines. Overall, ESWT could reverse the pathology of OA progression to some extent.

Both clinical trials and animal model studies have demonstrated the efficacy of ESWT for the treatment of OA. We summarized the potential role of ESWT in treating OA in a diagram (Figure 2). Specifically, ESWT has beneficial effects on cartilage, subchondral bone and surrounding tissues and can provide relief from chronic OA pain. However, the application of various ESWT methods has limited the quality of many existing studies. Future high-quality studies based on well-standardized parameters are needed, especially those reports focused on the effects on arthritic cartilage and subchondral bone, as well as pain symptom and motor function, which need to be further explored.

## Abbreviations

ACLT: anterior cruciate ligament transection
CGRP: calcitonin gene-related peptide
CTX-II: C-telopeptide of type II collagen
DRG: dorsal root ganglia
ERK: extracellular signal-regulated kinase
ESWT: extracorporeal shockwave therapy
FAK: focal adhesion kinase
MMP: matrix metalloproteinase
RA: rheumatoid arthritis
TLR3: Toll-like receptor 3
